# The contribution of work and health-related lifestyle to educational inequalities in physical health among older workers in Germany. A causal mediation analysis with data from the lidA cohort study

**DOI:** 10.1371/journal.pone.0285319

**Published:** 2023-08-09

**Authors:** Max Rohrbacher, Hans Martin Hasselhorn

**Affiliations:** Department of Occupational Health Science, School of Mechanical Engineering and Safety Engineering, University of Wuppertal, Wuppertal, Germany; Yonsei University College of Medicine, REPUBLIC OF KOREA

## Abstract

**Objectives:**

The objective of the study was to investigate the contribution of work factors and health-related lifestyle to educational inequalities in physical health among older workers in Germany by applying causal mediation analysis with longitudinal data.

**Methods:**

Data from the German lidA study was used. 2653 persons (53% female, 47% male) aged 46 (born 1965) and 52 (born 1959) at baseline were followed up for seven years with exposure and outcome assessments in 2011 (t0), 2014 (t1) and 2018 (t2). The total effect of education on physical health was decomposed into a natural direct effect (NDE) and a natural indirect effect (NIE) by using a sex-stratified causal mediation analysis with an inverse odds weighting approach. Baseline health, partner status and working hours were entered as a first set of mediators preceding the putative mediators of interest. All analyses were adjusted for age and migrant status.

**Results:**

Independent of the first set of mediators, work factors explained 21% of educational inequalities in physical health between low and high educated women and 0% comparing moderate versus high educated women. The addition of health behaviors explained further 26% (low vs. high education) and 20% (moderate vs. high education), respectively. Among men, net of the first set of mediators, work factors explained 5% of educational inequalities in physical health between low and high educated and 6% comparing moderate versus high educated persons. Additional 24% (low vs. high education) and 27% (moderate vs. high education) were explained by adding health behaviors to the models.

**Conclusions:**

To reduce educational inequalities in physical health among older workers in Germany, interventions to promote healthy behaviors are promising. Improving working conditions is likely an important prerequisite.

## Introduction

In many European countries the life expectancy is increasing and has contributed to demographic aging [1]. Driven by demographic aging, many European countries have adapted their social policies in order to extend the working life (EWL) [2, 3]. In Germany, early exits out of employment have increasingly been sanctioned, e.g. by restricting eligibility criteria for disability pension and by reducing the duration and amount of unemployment benefits; in parallel, the statutory retirement age has been raised and, continued employment has been incentivized [4]. These current EWL policies, which are mainly focused on (financial) pull-factors, unequally impact groups of different socioeconomic status (SES) [3, 4]. As opposed to high-skilled and high-earning workers at higher working age, those with lower SES have not benefitted equally from advances in health [1] and are therefore still at higher risk of health-related early exit from employment [5, 6]. Researchers therefore suggest that the combined effect of unequal health improvements and the unequal impact of retirement policies may aggravate existing health inequalities [7, 8].

### Social inequalities in health

Over the past decades, strong evidence of health inequalities in populations has accumulated. Most health complaints are more prevalent in lower SES groups and social inequalities in health are found at all ages with varying magnitude depending on the country [9, 10].

When examining health inequalities, the choice of the social status indicator as well as health indicator plays an important role. Emerging empirical evidence [11, 12] strongly suggests a longitudinal effect of the SES on health, supporting the causation hypothesis when older workers are investigated. However, these findings cannot be generalized to all health outcomes and the magnitude of the health gradient seems to depend on the operationalization of the SES [13].

Common operationalizations of the SES are education, occupation, and income [14]. Of the three, education may be the most suitable to investigate causal effects on health at higher working age. This is because scholarly education and vocational qualifications are obtained during the early career phase and the construct is very stable at higher working age [15]. Therefore, the assumption of temporal order of cause and effect is more easily fulfilled using education compared to current occupation and income.

While self-reported general health (SRH) and physical health exhibit gradients in the expected direction for all operationalizations of SES, this does not apply to common mental health disorders [13]. Studies using education to operationalize SES indicate weak and inconsistent associations between education and common mental health disorders [13, 16]. This may be one of the reasons why a number of existing studies on educational inequalities in health, focus on SRH as the outcome [cf. 11, 12]. SRH has been shown to be suitable for international comparison and to predict mortality and morbidity [17, 18]. However, there are multiple reasons why the (further) investigation of educational inequalities in physical health, rather than SRH, could be of interest, especially when examining older workers in Germany:

- Firstly, SRH and physical health are strongly related [19], but the latter may be a more objective measure than SRH [20] and more specifically assessing physical functioning at work and limitations of activities of daily living–and thus allowing for more precise conclusions.
- Secondly, some authors highlight that social inequalities in health may be underestimated when SRH is used as the outcome, because the association between health problems and SRH is stronger among higher educated persons [20].
- Lastly, in a previous study, educational differences in physical health explained a large proportion of social inequalities in early exit from employment [6]. Thus, investigating factors that contribute to physical health inequalities may provide further knowledge to extend the discussion of inequalities in early exits.

### Work, health-related lifestyle, and SES

Findings from studies based on cross-sectional as well as longitudinal data have stressed the role of work factors and health-related lifestyle contributing to health inequalities [11, 12, 21], thereby supporting the theoretical model on the explanation of health inequalities by Elkeles and Mielck [22]. A mediating role of work factors in the relationship between SES and health can be indirectly inferred from evidence indicating a higher prevalence of adverse working conditions among groups of low SES on the one hand, and evidence on the negative effects of adverse working conditions on health [23, 24] on the other hand. Physical demands are more prevalent among low-skilled individuals [11, 12, 21]. Similarly, lower skilled workers more often report a lack of resources such as influence at work, possibilities for development, job rewards and good leadership quality [11, 12, 21, 25]. However, an inverse gradient has been frequently observed for quantitative demands and efforts at work [6, 11, 26, 27].

With respect to health behaviors, there is a clear social gradient for smoking, leisure-time physical inactivity and high body weight, all more prevalent in lower SES groups [26, 28–30].

### Research need and study objectives

The assumption of mediation of the effect of SES on health by work factors and health behaviors is furthermore supported by a growing body of research applying mediation analysis [11]. However, longitudinal studies are still scarce and the mediation hypothesis has mainly been tested using a difference in coefficients approach which does not sufficiently take exposure-mediator interactions into account [see 11 for a systematic review of studies]. So far, to our knowledge, only three longitudinal studies have investigated the contribution of work factors and/or health behaviors to social inequalities in health among older workers [12, 31, 32]. Among these studies, the study by Schram et al. [12] was the first to simultaneously examine mediation by work factors and health behaviors. This was done by using causal mediation analysis. The study showed that among older European employees, 38% of the effect of low versus high education on SRH (RR 1.48, 95% CI 1.37–1.60) was mediated by working conditions (physical demands, lack of job control and lack of rewards combined) and 16% by health behaviors. However, their sensitivity analyses indicated that the effect mediation through working conditions and health behaviors varies by European region. Health behaviors contributed considerably more to health inequalities in Northern (Sweden, Denmark) and Western/Central Europe (Belgium, The Netherlands, France, Germany, Switzerland, Austria) compared to Southern countries (Greece, Spain, Italy) and the contribution of working conditions was large in Southern countries, lower in Western/Central Europe and non-existent in Northern countries. The study only contained a small subsample of older workers in Germany (around 10% of those from Western/Central European region) so that the specific contribution of work factors and health behaviors to health inequalities in Germany remains unclear.

To conclude, models and evidence indicate that work factors and health-related lifestyle mediate the effect of SES on health. This, however, has to our knowledge never been investigated longitudinally for older workers in Germany. The use of education as the SES indicator is suitable in this setting, given the stability of the construct at higher working age. Furthermore, as elaborated above, using physical health as the outcome may be advantageous in this context. Lastly, using causal mediation analysis with an Inverse Odds Weighting (IOW) approach [33] allows to study multiple mediators simultaneously, even in the presence of exposure-mediator interactions on the outcome [12, 34, 35].

Thus, the aim of the study is to investigate the contribution of work factors and health-related lifestyle to educational inequalities in physical health among older workers by applying causal mediation analysis with longitudinal data.

## Materials and methods

### Study design and participants

**Fig 1:**
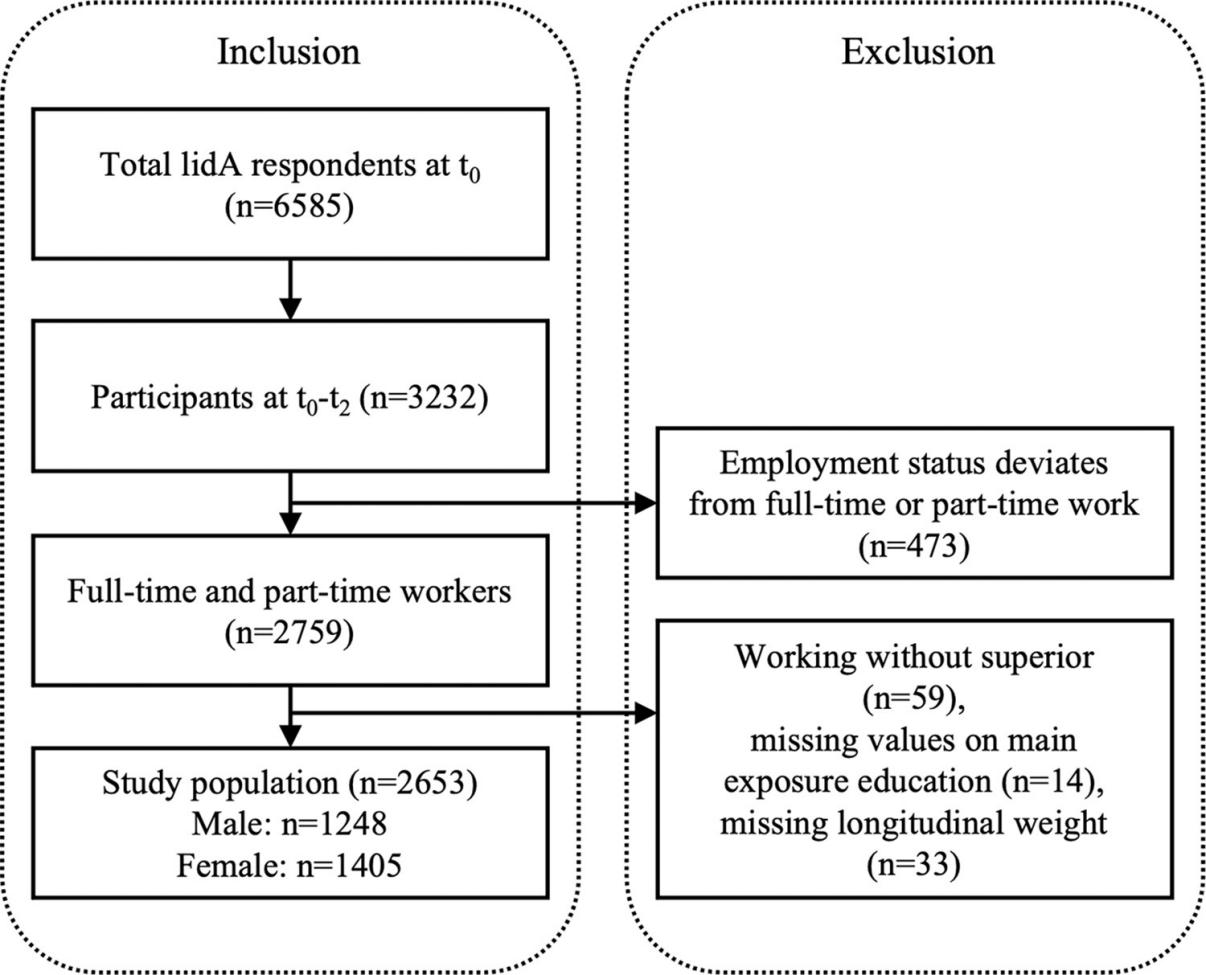
Summary of inclusion and exclusion criteria.

3232 subjects, who participated in all three existing waves (t0 = 2011, t1 = 2014, t2 = 2018) of the German lidA-study, were eligible for inclusion in the current study. lidA is a prospective cohort study investigating the topics, work, age, health, and labor market participation among persons from the German baby boom generation. Persons from two birth cohorts (born 1959 and 1965) have been interviewed every 3 or 4 years (2014, N = 4244; 2018, N = 3586) by computer assisted personal interviews (CAPI) given their initial participation in 2011 (N = 6585). The primary response rate, which is the ratio of achieved interviews at wave 1 (t0) (n = 6,637) to the operational sample (n = 24,322) was 27.3%. 6585 valid interviews were achieved. Descriptive statistics revealed only small deviations between the gross sample and respondents [36]. All outcome rates reported in line with the standard definitions by the American Association for Public Opinion Research (AAPOR), can be found in a methods report [36] and are similar to other representative employee surveys, e.g. the German Study on Mental Health at Work (S-MGA) [37].The lidA study is representative of older socially insured employees of the two cohorts with respect to sociodemographic characteristics, such as sex, education, and occupation [36, 38]. Self-employed and sworn civil servants were not included in the study. Further information on the study design can be found elsewhere [36, 38]. As can be seen in Fig 1, those with an employment status other than full-time or part-time were excluded from the sample, in order to assume similar exposure time to working conditions across the sample. Additionally, those without a superior were excluded, since two of the investigated covariates included assessments of the superior. Lastly, cases with missing values on the main exposure (education) were excluded. To account for potential selection bias a longitudinal non-response weight was calculated, which is described in further detail below. Subjects for which the weight could not be computed (n = 33) were also excluded, resulting in a final study sample of N = 2653 (53% female, 47% male).

All data was collected in accordance with the Declaration of Helsinki (1964) and its later amendments. Participants were informed about the aims and procedures of the study. Participation required oral consent at each study wave. The Ethics Committee of the University of Wuppertal approved the protocol for the lidA Cohort study [5 December 2008 (Sch/Ei Hasselhorn) and 20 November 2017 (MS/BB 171025 Hasselhorn)].

### Measurements

**Fig 2:**
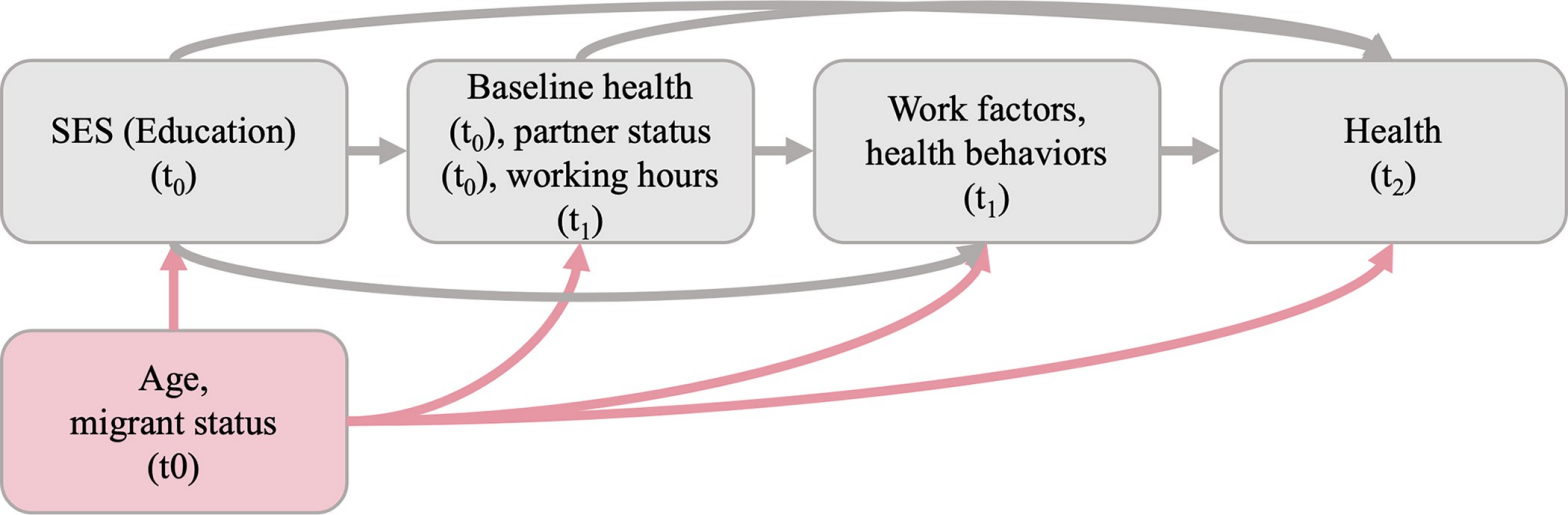
Assumed causal model for sex-stratified analysis.

In Fig 2 the assumed causal model is presented with confounders depicted in red boxes. “t0” indicates baseline assessment in 2011, “t1” first follow up in 2014 and “t2” second follow up in 2018. To ensure that all focal mediators succeeded the exposure and preceded the outcome, mediators were measured at t1, the exposure was measured at t0 and the outcome at t2. Further post-exposure variables, namely baseline health, partner status and working hours, which likely precede the focal mediators, were entered into the model as described in the section on statistical analyses and were measured either at t0 or t1.

### Physical health (t2)

Physical health was measured using the Short Form Health Survey (SF-12 PCS) (e.g. limitations “climbing several flights of stairs”, “[…] limited in kind of work or other daily activities […] as a result of your physical health”) [39, 40 p. 90–91]. A sum score ranging from 0 to 100 was computed following the procedure described by Nübling et al. [41]. The sum score was subsequently dichotomized as the Shapiro-Francia test indicated a significant deviation from normal distribution (V’ = 41.011, p-value >0.01). The inspection of normality tests and diagnostic plots showed that the distribution was moderately left skewed, with skewness of -0.71 at baseline (t0) and -0.53 at follow-up (t2). We used the 25^th^ percentile at t0 to determine subjects with poor physical health, which corresponds to a cut-off of 45 on a scale from 0–100. Previous studies have also used the 25^th^ percentile as cut-off to determine a poor physical health status [42]. Values of 50 or less on the SF-12 PCS scale may be indicative of a physical condition [40].

### Educational attainment (t0)

Our main independent variable was the educational level, which combined education and vocational training [43]. We trichotomized the variable into the three categories low (primary, lower secondary and upper secondary general education, cf. ISCED-97 1-3A), moderate (upper secondary vocational education and post-secondary non tertiary education, cf. ISCED-97 3B-4A), and high education (tertiary education, cf. ISCED-97 5–6).

### Baseline health, partner status and working hours (t0, t1)

Baseline physical health (t0), partner status (not single/single) (t0) and working hours (full-time/part-time) (t1) were entered as a first set of mediators to precede work factors and health-related lifestyle. The sequential approach is explained in the section about the statistical analysis below.

### Work factors (t1)

Time exposed to awkward body postures, heavy lifting, and repetitive movements was used to assess physical demands at work. Exposure to any of these dimensions for more than 25% of working hours was considered as high physical demands and exposure below that limit as low physical demands.

Influence at work, possibilities for development and leadership quality were each measured with three items from the German version of the Copenhagen Psychosocial Questionnaire (COPSOQ II, middle version) [44] and subsequently transformed into three separate sum scores ranging from 0–100. In this study, high scores indicate adverse exposure. Validation of the COPSOQ questionnaire in Germany indicated good psychometric properties of the single scales [44].

Rewards at work were measured by the reward short scale from the ERI instrument [45]. The short scale combines seven items (e.g., “I receive the respect I deserve from my superior or a respective relevant person”) to a total score ranging from 7.00 to 28.00. For the current analysis, the sum score was inverted so that a value of 28.00 reflects a total absence of rewards.

### Health-related lifestyle (t1)

Leisure-time physical activity, smoking and BMI were included as health-related lifestyles. Leisure-time physical activity was initially assessed with four response categories and dichotomized for the current analysis. Answer categories “I read, watch TV and do jobs that don’t involve much movement and are not physically demanding” and “I walk, cycle or exercise at least 4 hours a week. This includes walking, light gardening, but not commuting to work.” were recoded into “no or light physical activity”. Answer categories “I do at least 3 hours of physical exercise per week such as running, jogging, gymnastics, swimming, ball games, heavy gardening or other heavy physical activities.” and “I do intensive sports several times a week like running, swimming, cycling.” were recoded into “moderate or high physical activity”. Smoking was recoded into three categories dividing the sample into “non-smokers”, “former smokers” and “current smokers”. The BMI (weight in kg / body height in m^2^) was trichotomized into “normal (< 25)”, “overweight (≥ 25-< 30)” and “obese (≥30)”.

### Confounder

Age at baseline (46 [born 1965] or 52 [born 1959]) and migrant status (non-migrant/migrant) were used as the minimal adjustment set to calculate TE, NDE and NIE. Age and migrant status constitute background risks for physical health. Adjustment might reduce confounding.

### Statistical analysis

First descriptive statistics were displayed. All analyses were sex-stratified because previous analyses had shown the differentiated effect of work factors and health behaviors on health among women and men [9, 32, 46].

To investigate mediation effects we conducted a causal mediation analysis with inverse odds weighting [33]. The Total Effect (TE) of the main exposure (education) on physical health was decomposed into a Natural Direct Effect (NDE) and Natural Indirect Effect (NIE). NDE describes the effect of the exposure on the outcome with the pathway through the investigated intermediate(s) being deactivated [34, 35]. We note that the interpretation of the NDE is always relative to the mediators under study. The NIE describes the effect of the exposure on the outcome through the studied mediator(s) (without the direct effect element) [35, 47]. To account for potential selection bias, we computed a longitudinal non-response weight. The weight consists of two components, first a cross-sectional post-stratification weight and secondly, a non-response weight. Both components were multiplied to create a longitudinal weight:

$$Longitudinal\;weight={weight}_{t0}\times {weight}_{selected}$$

The post-stratification weight (weight_t0_) accounts for unequal selection probability by design and unequal participation probability at baseline (t_0_). For this weight an inverse probability weighting (IPW) was used for multiple socio-demographic variables including age, education, nationality and place of residence based on model-based selectivity analysis [36]. More detailed information about the procedure can be found elsewhere [36]. For selection into our final analysis sample, which only includes participants of all three waves, working full-time and part-time, with a superior (n = 2,653) we calculated a stabilized IPW (weight_selected_) [c.f. 48] for the variables age, education and migrant status. Mean weighting factors by educational level and sex are provided in the supplement (S1 Table).

To achieve the described effect decomposition, we followed the analysis steps described by Nguyen et al. [34]: First, an Inverse Odds Weight (IOW) was created by taking the inverse of the predicted log odds for each individual from an exposure model [34], which involved education as the dependent variable and the mediator(s) and confounders as independent variables. We used multinomial logistic regression for the exposure model. Subsequently, individuals from the exposed group (first the low educated, then the moderately educated) were assigned the IOW multiplied with the longitudinal non-response weight, while the reference group was assigned the longitudinal non-response weight only. The TE was then calculated using a general linear model with Poisson distribution, log link function and robust error variance [34, 49]. This is preferred over logistic regression in the presence of common (> 10%) outcomes to avoid an overestimation of the NDE [50, 51]. The NDE was calculated with the same model but including the IOW. This way the mediator is never entered into either of the outcome models. The NIE was calculated by subtracting the NDE from the TE. Using the following formula for ratio measures [52], the proportion of the TE mediated was then calculated for the relative risks (RR):

$$Proportion\;mediated\;(PM)=\frac{{RR}^{NDE}\times ({RR}^{NIE}-1)}{\left({RR}^{NDE}\times {RR}^{NIE}-1\right)}$$

All effect estimates and 95% CIs were computed using 1000 bootstrap replications. The described steps were conducted for each mediator individually and for all mediators combined using Stata V15.1 (College Station, TX: StataCorp LLC). The Stata code for the procedure, which was based on influential previous studies [34, 50, 53], is provided as a supplement (S1 File). Missing values were handled by Multiple Imputation by Chained Equations [54]. In the total sample, 12.1% of all observations had at least one missing value, which was mainly attributable to missingness on the job rewards variable. Hence, we regarded 10 imputations as sufficient and efficient [cf. 54]. The imputation model included all analysis variables as well as self-perceived general health as auxiliary variable.

To identify NDE and NIE several assumptions have to be made: These are no unmeasured confounding of the 1) exposure-mediator relationship, 2) mediator-outcome relationship and 3) exposure-outcome relationship as well as 4) no exposure induced confounding of the mediator-outcome relationship [35]. Although the panel design of our study is an advantage to separate cause and effect, the temporal ordering of exposure, mediators and outcome also introduces further risk for violation of some of these assumptions. Especially the fourth assumption may be violated in the presence of variables that are affected by the exposure and precede the focal mediators. We have identified three candidate variables which are likely exposure-induced mediator-outcome confounders, namely baseline health, partner status and working hours. To prevent violation of assumption four by these variables we followed a sequential mediation approach [55] to jointly assess mediation through the focal mediators (work factors, health behaviors), dependent on these preceding post-exposure confounders, which now constituted a first set of variables to be included in the mediator vector, as suggested by VanderWeele et al. [56]. Sequentially, work factors were then added to the mediator vector in a second step and both work factors and health behaviors in the third step. Supplementary, we assessed the mediation effects for all putative mediators individually based on the first set of mediators, including baseline health (S2 and S3 Tables).

### Sensitivity analysis

We calculated mediational E-values [57] to assess how strong potential unobserved confounders would have to be associated with both, the mediators under investigation and the outcome conditional on the measured confounders to explain away the NIE and to shift the 95% CI to include a relative risk (RR) of 1. This is a mediational analogue to the E-value introduced by VanderWeele et al. [57, 58] and can be calculated as follows, based on the observed RR for the NIE:

$$Mediational\;E-value={RR}^{obs}+\sqrt{{RR}^{obs}\times ({RR}^{obs}-1)}$$

## Results

Tables 1 and 2 show the characteristics of the female and male study sample, respectively. Among both women and men, those with low education had a higher prevalence of poor physical health at baseline and follow-up. The social gradient in physical health was stronger among men. However, relatively more women with low education compared to those with high education changed from having good health at baseline to poor health at follow up, which amplified the gradient at follow-up among women. The social gradient among men remained relatively stable. Regarding work factors a clear social gradient in the expected direction was observed for physical demands and possibilities for development among women (Table 1) and for physical demands among men (Table 2). Regarding health-related lifestyles the social gradient in BMI stood out among women (Table 1) and in smoking and leisure-time physical activity among men (Table 2).

**Table 1:** Characteristics of the female study sample by level of education before multiple imputation (n = 1 405).

Characteristics	**Education**			
	**low**	**moderate**	**high**	
Total n = 1405	**n = 227**	**n = 894**	**n = 284**	Missing values
	**n(%) or M(SD)**	**n(%) or M(SD)**	**n(%) or M(SD)**	**n(%)**
**Age at baseline (t0)**				0
46 (born 1965)	92 (40.5%)	516 (57.7%)	154 (54.2%)	
52 (born 1959)	135 (59.5%)	378 (42.3%)	130 (45.8%)	
**Migrant status (t0)**				0
non-migrant	193 (85.0%)	789 (88.3%)	232 (81.7%)	
migrant	34 (15.0%)	105 (11.7%)	52 (18.3%)	
**Partner status (t0)**				5 (0.4%)
not single	180 (79.3%)	762 (85.6%)	250 (88.3%)	
single	47 (20.7%)	128 (14.4%)	33 (11.7%)	
**Working hours (t1)**				0
part-time (< 35 hours/week)	137 (60.4%)	473 (52.9%)	140 (49.3%)	
full-time	90 (39.6%)	421 (47.1%)	144 (50.7%)	
**Physical health (t0)**				22 (1.6%)
good	155 (69.5%)	626 (71.4%)	239 (84.5%)	
poor	68 (30.5%)	251 (28.6%)	44 (15.5%)	
**Physical health (t2)**				13 (0.9%)
good	127 (57.0%)	553 (62.5%)	215 (75.7%)	
poor	96 (43.0%)	332 (37.5%)	69 (24.3%)	
**Physical demands (t1)**				0
low	84 (37.0%)	413 (46.3%)	149 (52.5%)	
high	143 (63.0%)	480 (53.7%)	135 (47.5%)	
**Influence at work (t1)** 1 (highest)– 100 (lowest)	67.4 (26.2)	65.8 (25.3)	56.2 (25.2)	2 (0.1%)
**Possibilities for development (t1)** 1 (highest)– 100 (lowest)	43.9 (23.6)	36.6 (19.6)	30.9 (19.6)	0
**Leadership Quality (t1)** 1 (highest)– 100 (lowest)	40.7 (20.7)	41.7 (20.0)	41.6 (20.0)	13 (0.9%)
**Rewards at work (t1)** 7 (highest)– 28 (lowest)	10.1 (3.9)	10.3 (3.6)	10.6 (3.4)	134 (9.5%)
**BMI (t1)**				38 (2.7%)
normal	68 (30.4%)	363 (42.0%)	141 (50.5%)	
overweight	84 (37.5%)	318 (36.8%)	99 (35.5%)	
obese	72 (32.1%)	183 (21.2%)	39 (14.0%)	
**Smoker (t1)**				1 (0.1%)
never	80 (35.2%)	379 (42.4%)	159 (56.0%)	
former	66 (29.1%)	266 (29.8%)	65 (22.9%)	
current	81 (35.7%)	248 (27.8%)	60 (21.1%)	
**Leisure-time physical activity (t1)**				0
moderate or high PA	66 (29.1%)	318 (35.6%)	114 (40.1%)	
no or light PA	161 (70.9%)	576 (64.4%)	170 (59.9%)	

Valid column percentages displayed; Data are presented as mean (SD) for continuous measures, and n (%) for categorical measures.

**Table 2:** Characteristics of the male study sample by level of education before multiple imputation (N = 1 248).

Characteristics	**Education**			
	**low**	**moderate**	**high**	
Total n = 1248	**n = 298**	**n = 615**	**n = 335**	Missing values
	**n(%) or M(SD)**	**n(%) or M(SD)**	**n(%) or M(SD)**	**n(%)**
**Age at baseline (t0)**				0
46 (born 1965)	158 (53.0%)	346 (56.3%)	192 (57.3%)	
52 (born 1959)	140 (47.0%)	269 (43.7%)	143 (42.7%)	
**Migrant status (t0)**				
non-migrant	245 (82.2%)	528 (85.9%)	287 (85.7%)	
migrant	53 (17.8%)	87 (14.1%)	48 (14.3%)	
**Partner status (t0)**				3 (0.2%)
not single	259 (86.9%)	558 (91.0%)	308 (92.2%)	
single	39 (13.1%)	55 (9.0%)	26 (7.8%)	
**Working hours (t1)**				0
part-time (< 35 hours/week)	5 (1.7%)	14 (2.3%)	21 (6.3%)	
full-time	293 (98.3%)	601 (97.7%)	314 (93.7%)	
**Physical health (t0)**				11 (0.9%)
good	178 (60.3%)	471 (77.6%)	294 (88.6%)	
poor	117 (39.7%)	136 (22.4%)	38 (11.4%)	
**Physical health (t2)**				16 (1.3%)
good	151 (52.2%)	416 (68.3%)	269 (80.5%)	
poor	138 (47.8%)	193 (31.7%)	65 (19.5%)	
**Physical demands (t1)**				0
low	133 (44.6%)	337 (54.8%)	226 (67.5%)	
high	165 (55.4%)	278 (45.2%)	109 (32.5%)	
**Influence at work (t1)** 1 (highest)– 100 (lowest)	59.3 (27.9)	59.1 (26.5)	51.2 (23.6)	0
**Possibilities for development (t1)** 1 (highest)– 100 (lowest)	38.8 (19.0)	35.0 (19.3)	31.3 (18.4)	0
**Leadership Quality** 1 (highest)– 100 (lowest)	40.4 (18.8)	41.6 (18.6)	42.8 (18.0)	10 (0.8%)
**Rewards at work (t1)** 7 (highest)– 28 (lowest)	10.1 (3.5)	9.9 (3.3)	9.7 (3.2)	83 (6.6%)
**BMI (t1)**				5 (0.4%)
normal	39 (13.1%)	111 (18.2%)	97 (29.2%)	
overweight	161 (54.0%)	332 (54.2%)	162 (48.6%)	
obese	98 (32.9%)	169 (27.6%)	74 (22.2%)	
**Smoker (t1)**				0
never	83 (27.9%)	220 (35.8%)	180 (53.7%)	
former	94 (31.5%)	208 (33.8%)	88 (26.3%)	
current	121 (40.6%)	187 (30.4%)	67 (20.0%)	
**Leisure-time physical activity (t1)**				1 (0.1%)
moderate or high PA	97 (32.6%)	244 (39.7%)	170 (50.9%)	
no or light PA	201 (67.4%)	371 (60.3%)	164 (49.1%)	

Valid column percentages displayed; Data are presented as mean (SD) for continuous measures, and n (%) for categorical measures.

In Table 3, results from the mediation analyses among the female subsample are displayed. Compared to women with high education, those with low education had a 1.55-fold (95% CI 1.42–1.68) and those with moderate education a 1.54-fold (95% CI 1.42–1.65) higher risk of reporting poor physical health at follow-up (total effects). Comparing low versus high educated women, the first set of mediators, including baseline health, partner status and working hours mediated 18% of the TE (RR^NIE^ 1.07, 95% CI 1.04–1.10). The second set of mediators, additionally including all work factors, explained 39% of the TE (RR^NIE^ 1.16, 95% CI 1.10–1.21). Hence, independent of the first set of mediators (39%-18%), all work factors combined explained 21% of the TE comparing low versus high educated. The complete set of mediators, additionally including all health behaviors, mediated 65% of the TE (RR^NIE^ 1.30, 95% CI 1.19–1.42). Thus, independent of work factors, baseline health, partner status and working hours, health behaviors explained 26% of the TE. Independent of the complete set of mediators the effect of low versus high education on physical health among women was RR^NDE^ 1.19 (95% CI 1.03–1.35).

**Table 3:** Decomposition of the total effect (TE) of education on physical health into natural direct effect (NDE) and natural indirect effect (NIE) using baseline health, work factors and health behaviors as mediators. Imputed female subsample (n = 1 405). Adjusted for age and migrant status.

	Low vs. high education			Moderate vs. high education		
	RR	95% CI^a^	PM^b^ %	RR	95% CI^a^	PM^b^ %
Total effect of education on physical health	1.55	1.42–1.68		1.54	1.42–1.65	
Mediation by baseline health^c^						
NIE	1.07	1.04–1.10	18	1.16	1.14–1.17	39
NDE	1.45	1.32–1.58		1.33	1.23–1.43	
Mediation by baseline health^c^ & work factors						
NIE	1.16	1.10–1.22	39	1.16	1.14–1.18	39
NDE	1.34	1.21–1.48		1.33	1.22–1.43	
Mediation by baseline health^c^ & work factors & health behaviors						
NIE	1.30	1.19–1.42	65	1.26	1.23–1.29	59
NDE	1.19	1.03–1.35		1.22	1.12–1.32	

^a^obtained from bootstrapping (1 000 reps); ^b^Proportion mediated (PM) = RR_NDE_*(RR_NIE_-1)/(RR_NDE_*RR_NIE_-1)

^*c*^plus partner status and working hours

[RR = relative risk; CI = confidence interval].

Comparing women with moderate versus high education, the first set of mediators mediated 39% of the TE (RR^NIE^ 1.16, 95% CI 1.14–1.17). The addition of all work factors to the first set of mediators did not increase the PM (RR^NIE^ 1.16, 95% CI 1.14–1.18). The complete set of mediators, additionally including all health behaviors, mediated 59% of the TE (RR^NIE^ 1.26, 95% CI 1.23–1.29). Hence, independent of all other mediators, health behaviors explained 20% of the TE comparing moderate versus high educated women. Independent of the complete set of mediators the effect of moderate versus high education on physical health among women was RR^NDE^ 1.22 (95% CI 1.12–1.32).

Additional analyses (S2 Table) of the female sub-sample investigating all work factors and all health behaviors individually dependent on the first set of mediators (baseline health, partner status, working hours), indicate that influence at work and possibilities for development (only between low and highly educated) as well as BMI and smoking (the latter only between moderate and highly educated) may be dominant mediators, showing the largest NIE.

Table 4 shows the results from the mediation analyses among the male subsample. Compared to men with high education, those with low education had a 2.14-fold (95% CI 1.96–2.32) and those with moderate education a 1.59-fold (95% CI 1.46–1.71) higher risk of reporting poor physical health at follow-up (total effects). Comparing low versus high educated men, the first set of mediators, namely baseline health, partner status and working hours, mediated 56% of the TE (RR^NIE^ 1.43, 95% CI 1.36–1.49). The magnitude of the mediated effects increased by 5% to 61% (RR^NIE^ 1.48, 95% CI 1.39–1.57) when all work factors were added to the first set of mediators. The complete set of mediators, additionally including all health behaviors, mediated 85% of the TE (RR^NIE^ 1.83, 95% CI 1.70–1.96) comparing low versus highly educated. Thus, independent of work factors, baseline health, partner status and working hours, health behaviors explained 24% of the TE. Independent of the complete set of mediators the effect of low versus high education on physical health among men was RR^NDE^ 1.17 (95% CI 1.03–1.31).

**Table 4:** Decomposition of the total effect (TE) of education on physical health into natural direct effect (NDE) and natural indirect effect (NIE) using baseline health, work factors and health behaviors as mediators. Imputed male subsample (n = 1 248). Adjusted for age and migrant status.

	Low vs. high education			Moderate vs. high education		
	RR	95% CI^a^	PM^b^ %	RR	95% CI^a^	PM^b^ %
Total effect of education on physical health	2.14	1.96–2.32		1.59	1.46–1.71	
Mediation by baseline health^c^						
NIE	1.43	1.36–1.49	56	1.13	1.11–1.15	31
NDE	1.50	1.30–1.70		1.40	1.28–1.52	
Mediation by baseline health^c^ & work factors						
NIE	1.48	1.39–1.57	61	1.16	1.13–1.19	37
NDE	1.45	1.30–1.61		1.37	1.25–1.49	
Mediation by baseline health^c^ & work factors & health behaviors						
NIE	1.83	1.70–1.96	85	1.31	1.26–1.35	64
NDE	1.17	1.03–1.31		1.21	1.10–1.32	

^a^obtained from bootstrapping (1 000 reps)

^b^Proportion mediated (PM) = RR_NDE_*(RR_NIE_-1)/(RR_NDE_*RR_NIE_-1)

^c^plus partner status and working hours

[RR = relative risk; CI = confidence interval]

Comparing men with moderate versus high education, the first set of mediators mediated 31% of the TE (RR^NIE^ 1.13, 95% CI 1.11–1.15). The second set of mediators, additionally including all work factors, explained 37% of the TE (RR^NIE^ 1.16, 95% CI 1.13–1.19). Thus, net of first set of mediators all work factors combined explained 6% of the TE comparing moderate versus high educated men. The complete set of mediators, additionally including all health behaviors, mediated 64% of the TE (RR^NIE^ 1.31, 95% CI 1.26–1.35). Thus, independent of all other mediators, health behaviors explained 27% of the TE. Independent of the complete set of mediators the effect of moderate versus high education on physical health was RR^NDE^ 1.21 (95% CI 1.10–1.32). Additional analyses (S3 Table) of the male sub-sample investigating all work factors and all health behaviors individually dependent on the first set of mediators (baseline health, partner status, working hours), indicate that BMI (only between moderate and highly educated), smoking and physical activity may be dominant mediators, showing the largest NIE.

### Sensitivity analysis

We calculated mediational E-values [57] for our main analyses displayed in Tables 3 and 4. Overall all mediational E-values took relatively high values (S4 and S5 Tables) indicating that moderate to strong unmeasured confounding would be necessary to explain away the observed NIE. The following form of statements was adopted from Smith et al. [57] and VanderWeele et al. [58]. Comparing low to high educated women (S4 Table), an unmeasured confounder associated with both the mediators (baseline health, work factors and health behaviors) and physical health with approximate RR of 1.92 each, above and beyond the measured covariates, would completely explain away the observed indirect effect, but weaker confounding could not (S4 Table). To shift the 95% CI to include a RR of 1 an unmeasured confounder associated with both, the mediators and physical health with an approximate RR of 1.67 each, above and beyond the measured covariates, could suffice, but weaker confounding could not (S4 Table). For the analysis comparing low versus high educated men, unmeasured confounding would have to be even stronger, with mediational E-values of RR 3.06 to explain away the NIE and RR 2.79 to shift the 95% CI to include a RR of 1 (S5 Table).

## Discussion

Our analysis showed that educational inequalities in physical health are present among older workers in Germany. Both low and moderately educated women had an approximate 1.5-fold higher risk of reporting poor physical health at follow-up compared to their highly educated counterparts. Among men these educational differences in physical health were slightly more accentuated with approximate 2-fold higher risk comparing low versus highly educated and approximate 1.6-fold risk comparing moderately versus highly educated persons.

Using a sex-stratified causal mediation analysis via inverse odds weighting, we could quantify the extent to which work factors and the health-related lifestyles mediate the effect of education on physical health, hence contribute to educational inequalities in physical health, dependent on baseline health, partner status and working hours (i.e. the first set of mediators) controlling for age and migrant status. Among women the first set of mediators explained 18% of the educational differences in physical health (i.e. the TE) between low and high educated. Independent of the first set of mediators, work factors could explain 21% of the TE. By addition of health behaviors, a further 26% of the TE could be explained. The full set of mediators explained 65% of the TE. Comparing moderate to high educated women the first set of mediators explained 39% of the TE. The magnitude of the mediated effect did not increase when adding work factors but increased by additional 20% by adding all health behaviors. The full set of mediators explained 59% of the TE. Among men the first set of mediators already explained 56% of the educational differences in physical health between low and highly educated. Additional 5% of the TE was explained by including work factors and additional 24% by also including health behaviors. The full set of mediators explained 85% of the TE. Comparing moderate to high educated men, the first set of mediators explained 31% of the TE. Work factors added 6% to the proportion mediated and health behaviors additional 27%. The full set of mediators explained 59% of the TE.

### Comparison with existing evidence

Supporting current evidence on social inequalities in health, our study showed that both work factors and health behaviors contribute to health inequalities [11, 12]. Unlike the few existing studies examining the contribution of work factors and/or health behaviors to social inequalities in health among older workers with longitudinal data [12, 31, 32], we used the SF-12 physical component summary [39] to assess physical health as the outcome.

In a previous systematic review, Dieker et al. [11] reported that work factors explained 14–38% of social inequalities in self-rated health in longitudinal studies. This aligns with our study only with respect to physical health inequalities between low and highly educated women, where, independent of baseline health and other post-exposure variables, work factors explained 21% of physical health inequalities. Among men this contribution amounted to maximum 6%. Also, when compared to recent findings by Schram et al. [12] using longitudinal SHARE (Survey of Health, Ageing, and Retirement in Europe) data from 16 countries, our results indicate a much smaller contribution of work factors and a larger contribution of health behaviors to health inequalities. This may have several reasons related to the measurement of exposure and outcome, differences concerning the analytical approaches and cross-country differences in the contribution of work factors and health behaviors to health inequalities.

First, the weaker contribution of work factors could be due to our choice of the exposure–education may reflect health behaviors more strongly than working conditions [13]–and our choice of the outcome–health behaviors may be more important than work factors in explaining inequalities in physical health [59]. Although this was an unexpected result, some authors report similar findings with respect to the contribution of work factors. For example Schmitz [31] concluded that “[…] baseline health, educational attainment, and mid-career earnings growth […] exert a stronger influence on health at older ages than current job demands […]” [31, p.18]. Similarly, Warren et al. [32] found that job characteristics only contributed to a SRH decline among women but not among men. Also, in our study the contribution of work factors to educational inequalities in health was largest among women. A further possible explanation could be that the effect of psychosocial work factors–four out of five work factors in our analysis may be regarded as such–on physical health is time-delayed and cumulative, perhaps even mediated through health behaviors (e.g., smoking, physical activity, body weight) whose influence on physical functioning is more immediate. This assumption is also supported by findings from studies applying a life course perspective to examine the effects of work-related stress on physical functioning [60, 61]. Yet another explanation, in line with Schmitz [31], could be that the effects of education and baseline health overscore the influence of the current job demands on health among older workers.

Hence, a second major explanation of our study results in comparison to previous findings could be that by including the baseline physical health status in a first set of mediators preceding working conditions, past exposures to adverse working conditions may be reflected by the initial health status with which participants entered the study. Therefore, the addition of current working conditions may have contributed less to health inequalities than expected.

Finally, with respect to the contribution of the investigated mediators to educational inequalities in health, cross-country differences are important to note. E.g., our findings correspond to some degree to a sensitivity analysis provided by Schram et al. [12], where the contributions of work factors and health behaviors were investigated stratified by European region. Similar to their findings for Northern (37%) and Western (31%) countries we found that health-related lifestyles explained 26% of health inequalities between low versus high educated women and 24% between low versus high educated men. In our study however, this proportion can be interpreted as the contribution of health behaviors independent of work factors and baseline health. With respect to the contribution of work factors, our findings deviate from those for Western countries (including a subsample of German older workers) in the study by Schram et al. [12] but are in fact similar to their findings for Northern countries, where work factors seem to play a minor role in explaining educational inequalities in health [12]. In our study this mainly applies to the inequalities among men. The contribution of work factors was larger comparing low versus high educated women.

Findings from our additional analyses (S2 and S3 Tables) furthermore corroborate previous cross-sectional findings which indicated that educational inequalities in smoking are more pronounced among men, and inequalities in overweight and obesity are more pronounced among women [9]. The additional analyses also showed that among women, out of all investigated work factors, possibilities for development contributed by far the most to educational inequalities in physical health when analyzed separately, dependent on baseline health. This also aligns with previous cross-sectional findings on occupational inequalities in (mental) health [21, 25].

### Strengths and limitations

This study adds to the existing evidence in multiple ways. To our knowledge this is the first longitudinal study among older workers in Germany applying a causal mediation analysis to investigate the contribution of physical and psychosocial work factors as well as health-related lifestyles to educational inequalities in physical health. One of the strengths is the narrow focus on older workers of the German baby boom cohorts, 1959 and 1965. We found that our results differed from those of a previous study investigating (self-rated) health inequalities in European regions [12], especially with respect to the contribution of work factors. This implies that the explanation of health inequalities may not only vary between European regions (Northern, Western/Central, Southern, Eastern) but also strongly within those regions and in dependence of deployed health outcomes. Hence, our findings may not be applicable to other countries.

A further strength is the use of specific indicators of the SES and health as well as providing a rationale for this choice. In the past, authors have repeatedly stressed that measures of the SES [13] as well as health [13, 62] are not interchangeable and that the pathways between SES and health may vary depending on the selected indicators [13]. The contribution of the investigated intermediates to physical health inequalities may vary depending on the selection of the SES indicator. E.g., by using occupational class as the main exposure, we assume that work factors would gain more importance as mediators. This however comes with a greater risk of reverse causation.

Using the IOW approach, we were able to investigate multiple mediators simultaneously irrespective of a possible exposure-mediator interaction on the outcome, which we regard as a strength, especially when assessing SES indicators as the main exposure. Nonetheless, the application of causal mediation analysis is accompanied by several no-confounding assumptions. The panel design of our study is a strength and allows to temporally order exposure (X), mediators (M), and outcome (Y). However, inherent with the temporal order of the study variables is the risk of exposure-induced confounding of the mediator-outcome association. Importantly, studying health inequalities among older workers requires consideration of the initial health status with which participants entered the study, but this baseline health is itself affected by education (X) and has effects on M and Y. Therefore, to prevent violation of the assumption about the absence of exposure-induced confounding, we included baseline health in a first set of mediators. We then followed a sequential mediation approach to jointly assess mediation through the focal mediators (M) dependent on this first set of mediators [55, 56]. The joint assessment changed the causal diagram in a way that there is no more confounder of the mediator-outcome association affected by the exposure [56], but rather the effect of the exposure is assumed to mediate through both, the first set of mediators including baseline health and the focal mediators or through either of them. While this approach allows us to identify NDE and NIE even in the presence of an exposure-induced mediator-outcome confounder, strong no-unmeasured confounding assumptions are still necessary for the causal interpretation of the results [56]. For the so called sequential ignorability assumption [63] to hold, the absence of unmeasured confounding of exposure-mediator, mediator-outcome, and exposure-outcome associations is required. These however are strong assumptions, even in settings where the treatment/exposure can be randomized [33] as residual unobserved confounding of the mediators and the outcome cannot be ruled out. Many researchers therefore strongly recommend conducting sensitivity analysis to examine the impact of unobserved confounding [52, 57, 63, 64]. As data on unmeasured confounding is by definition unavailable, most available methods require the investigators to specify a large number of sensitivity-analysis parameters [56] and re-run the mediation analyses under varying levels of unobserved confounding. This is sometimes difficult to interpret in practice and also computationally demanding, especially when investigating multiple mediators with multiply imputed data. We therefore calculated mediational E-values as an approximate sensitivity analysis for unmeasured mediator-outcome confounding as suggested by Smith et al. [57] in order to quantify how strong unobserved confounding would have to be to explain away the indirect effects. We found that rather strong confounding would be necessary for all the main analyses, above and beyond the measured covariates, in order to explain away the NIE or shift the 95% CI to include a RR of 1. We are, however, not aware of specific variables that could take these values, which were not already accounted for.

Lastly, selective dropout of participants during follow-up and the application of several inclusion criteria may have introduced selection bias. To address potential selection bias, a longitudinal non-response weight was used.

### Implications

From an ethical perspective the findings provide reasons for discussion because the control over health-related lifestyles is first and foremost in the hands of the workers themselves. Nonetheless, one may argue that improvements of working conditions may also lead to healthier lifestyles. A previous study showed that adverse working conditions and unhealthy behaviors cluster among lower educated individuals [65]. Hence, the existence of work-related risk factors may increase the occurrence and effect of lifestyle-related risk factors and vice versa. (Night) shift work constitutes a prime example for the interaction of work factors and health behaviors. It affects health directly, through the desynchronization of the circadian rhythm and indirectly, through social desynchronization and health-related lifestyle changes [66]. Aside from this example, a meta-analysis by Heikkilä et al. [67] also provides evidence of the association between psychosocial work stress and the co-occurrence of unhealthy lifestyles, including overweight, current smoking and physical inactivity during leisure time.

Therefore, interventions promoting healthy behaviors should go hand in hand with interventions targeting adverse working conditions. Our findings suggest that promoting healthy behaviors, if targeted specifically at low and moderately educated, may be a promising approach to reduce educational inequalities in physical health among older workers in Germany. A reduction of physical health inequalities may in turn help to counteract a widening of social inequalities in early exits into long-term unemployment [6]. Secondly, educational inequalities in physical health could be reduced by improving working conditions. Especially low educated women should be targeted. Independent of baseline health, partner status and working hours, work factors accounted for 21% of physical health inequalities between low and highly educated women. Separate analyses indicate that those with lower education should be granted improved possibilities for development in order to reduce existing inequalities.

Furthermore, from a scientific standpoint, it again becomes apparent that a life course perspective should be adopted when investigating health inequalities. Educational inequalities in physical health during the late working career may likely not be fully explained by older workers’ current circumstances alone. In the present study, especially among men, the first set of mediators, including baseline health, already explained more than 50% of the educational inequalities in health at follow-up between low and high educated persons. Thus, education, but also the baseline health status of participants may reflect the accumulation of health risks during the many preceding years in multiple (dis-)advantaging contexts [cf. 68, cf. 69]. This implies on the one hand that, where possible, data on determinants of health should be collected starting early in life. On the other hand, in the many cases where this is not possible due to time-, financial and data-protection- constraints, researchers should continue to discuss the consequences of their choices of SES indicators and health outcomes on the generalizability of their findings. For future studies investigating the contribution of work factors and health-related lifestyles to physical health inequalities among older workers, we recommend the (additional) use of different SES indicators, such as the occupational class, to examine how the importance of the intermediates may change.

## Conclusion

Work factors and the health-related lifestyle contribute to educational inequalities in physical health among older workers in Germany. The findings indicate that health-related lifestyles are more powerful to explain educational inequalities in physical health than work factors among both sexes, possibly due to the choice of our SES indicator, the choice of the health outcome and the time-delayed and cumulative effect of work factors on physical functioning. To attenuate these health inequalities, interventions promoting healthy behaviors should go hand in hand with interventions addressing adverse working conditions and should be targeted specifically at lower and moderately educated persons. Additional analyses suggest that among women, BMI and possibilities for development, and among men, smoking and physical activity constitute key variables to target.

## Supporting information

S1 Checklist STROBE statement—Checklist of items that should be included in reports of *cohort studies*. (PDF)

S1 Table Mean weighting factors by education and sex. (DOCX)

S2 Table Decomposition of the total effect (TE) of education on physical health into natural direct effect (NDE) and natural indirect effect (NIE) using baseline health, work factors and health behaviors individually as mediators. Imputed female subsample (n = 1 405). TE and significant NIE and respective proportion mediated (PM) marked in bold. Adjusted for age and migrant status. [RR = relative risk; CI = confidence interval].
(DOCX)

S3 Table Decomposition of the total effect (TE) of education on physical health into natural direct effect (NDE) and natural indirect effect (NIE) using baseline health, work factors and health behaviors individually as mediators. Imputed male subsample (n = 1 248). TE and significant NIE and respective proportion mediated (PM) marked in bold. Adjusted for age and migrant status. [RR = relative risk; CI = confidence interval].
(DOCX)

S4 Table Mediational E-values for the female sub-sample to quantify the minimum strength of the association that an unmeasured confounder would need to have with both the outcome and the mediators to fully explain away the NIE. (DOCX)

S5 Table Mediational E-values for the male sub-sample to quantify the minimum strength of the association that an unmeasured confounder would need to have with both the outcome and the mediators to fully explain away the NIE. (DOCX)

S1 File Stata code for mediation analysis using inverse odds weighting with imputed data; example for comparing low versus high educated men using all putative mediators. (PDF)
