# Supplementary material for: The contribution of work and health-related lifestyle to educational inequalities in physical health among older workers in Germany. A causal mediation analysis with data from the lidA cohort study
Source: PLoS One. 2023 Aug 9;18(8):e0285319. doi: 10.1371/journal.pone.0285319 (PMC10411755; doi:10.1371/journal.pone.0285319)
Supplement: S1 Table — (DOCX) [file pone.0285319.s002.docx]

**S1 Table. Mean weighting factors by education and sex**

|  | education | | |
| --- | --- | --- | --- |
| Sex | low | moderate | high |
| male | 1.293 | 0.910 | 0.833 |
| female | 1.285 | 0.952 | 0.880 |
