# Supplementary material for: The contribution of work and health-related lifestyle to educational inequalities in physical health among older workers in Germany. A causal mediation analysis with data from the lidA cohort study
Source: PLoS One. 2023 Aug 9;18(8):e0285319. doi: 10.1371/journal.pone.0285319 (PMC10411755; doi:10.1371/journal.pone.0285319)
Supplement: S2 Table — TE and significant NIE and respective proportion mediated (PM) marked in bold. Adjusted for age and migrant status. [RR = relative risk; CI = confidence interval]. (DOCX) [file pone.0285319.s003.docx]

***S2. D*ecomposition of the total effect (TE) of education on physical health into natural direct effect (NDE) and natural indirect effect (NIE) using *baseline health,* work factors and health behaviors *individually* as mediators. Imputed female subsample (n=1 405).** Adjusted for age *and migrant status*. [RR = relative risk; CI = confidence interval]

|  | Low vs. high education | | |  | Moderate vs. high education | | |
| --- | --- | --- | --- | --- | --- | --- | --- |
|  | RR | 95% CI^a^ | PM^b^ % |  | RR | 95% CI^a^ | PM^b^ % |
| Total effect of education on physical health | 1.55 | 1.42-1.69 |  |  | 1.54 | 1.42-1.65 |  |
| Mediation by baseline health^c^ |  |  |  |  |  |  |  |
| NIE | 1.07 | 1.04-1.11 | 18 |  | 1.16 | 1.14-1.17 | 39 |
| NDE | 1.45 | 1.31-1.58 |  |  | 1.33 | 1.23-1.43 |  |
| Analysis 1: baseline health* & physical demands |  |  |  |  |  |  |  |
| NIE | 1.08 | 1.04-1.12 | 21 |  | 1.17 | 1.15-1.19 | 42 |
| NDE | 1.43 | 1.3-1.57 |  |  | 1.31 | 1.21-1.41 |  |
| Analysis 2: baseline health* & influence at work |  |  |  |  |  |  |  |
| NIE | 1.12 | 1.07-1.17 | 30 |  | 1.14 | 1.12-1.16 | 35 |
| NDE | 1.39 | 1.25-1.52 |  |  | 1.35 | 1.24-1.45 |  |
| Analysis 3: baseline health* & possibilities for development |  |  |  |  |  |  |  |
| NIE | 1.19 | 1.13-1.26 | 45 |  | 1.17 | 1.15-1.19 | 42 |
| NDE | 1.30 | 1.16-1.44 |  |  | 1.31 | 1.21-1.41 |  |
| Analysis 4: baseline health* & leadership quality |  |  |  |  |  |  |  |
| NIE | 1.07 | 1.03-1.10 | 18 |  | 1.16 | 1.14-1.17 | 39 |
| NDE | 1.45 | 1.32-1.59 |  |  | 1.33 | 1.22-1.43 |  |
| Analysis 5: baseline health* & rewards |  |  |  |  |  |  |  |
| NIE | 1.05 | 1.02-1.09 | 14 |  | 1.15 | 1.13-1.17 | 37 |
| NDE | 1.47 | 1.34-1.61 |  |  | 1.34 | 1.23-1.44 |  |
| Analysis 6: baseline health* & BMI |  |  |  |  |  |  |  |
| NIE | 1.28 | 1.22-1.33 | 61 |  | 1.18 | 1.16-1.20 | 44 |
| NDE | 1.22 | 1.10-1.33 |  |  | 1.30 | 1.20-1.40 |  |
| Analysis 7: baseline health* & smoking |  |  |  |  |  |  |  |
| NIE | 1.04 | 1.00-1.08 | 11 |  | 1.21 | 1.19-1.24 | 50 |
| NDE | 1.49 | 1.34-1.63 |  |  | 1.27 | 1.17-1.36 |  |
| Analysis 8: baseline health* & physical activity |  |  |  |  |  |  |  |
| NIE | 1.09 | 1.06-1.13 | 23 |  | 1.16 | 1.15-1.18 | 40 |
| NDE | 1.42 | 1.29-1.55 |  |  | 1.32 | 1.22-1.42 |  |
| ^a^obtained from bootstrapping (1 000 reps); ^b^Proportion mediated (PM) = RR_NDE_*(RR_NIE_-1)/(RR_NDE_*RR_NIE_-1)  ^c^plus partner status and working hours | | | | | | | |
