# Supplementary material for: The contribution of work and health-related lifestyle to educational inequalities in physical health among older workers in Germany. A causal mediation analysis with data from the lidA cohort study
Source: PLoS One. 2023 Aug 9;18(8):e0285319. doi: 10.1371/journal.pone.0285319 (PMC10411755; doi:10.1371/journal.pone.0285319)
Supplement: S3 Table — Imputed male subsample (n = 1 248). TE and significant NIE and respective proportion mediated (PM) marked in bold. Adjusted for age and migrant status. [RR = relative risk; CI = confidence interval]. (DOCX) [file pone.0285319.s004.docx]

**S3. Decomposition of the total effect (TE) of education on physical health into natural direct effect (NDE) and natural indirect effect (NIE) using baseline health, work factors and health behaviors individually as mediators. Imputed male subsample (n=1 248).** Adjusted for age and migrant status. [RR = relative risk; CI = confidence interval]

|  | Low vs. high education | | |  | Moderate vs. high education | | |
| --- | --- | --- | --- | --- | --- | --- | --- |
|  | RR | 95% CI^a^ | PM^b^ % |  | RR | 95% CI^a^ | PM^b^ % |
| Total effect of education on physical health | 2.14 | 1.96-2.32 |  |  | 1.59 | 1.46-1.71 |  |
| Mediation by baseline health^c^ |  |  |  |  |  |  |  |
| NIE | 1.43 | 1.36-1.49 | 56 |  | 1.13 | 1.11-1.15 | 31 |
| NDE | 1.50 | 1.30-1.70 |  |  | 1.40 | 1.28-1.52 |  |
| Analysis 1: baseline health^c^ & physical demands |  |  |  |  |  |  |  |
| NIE | 1.42 | 1.35-1.49 | 55 |  | 1.15 | 1.13-1.18 | 36 |
| NDE | 1.51 | 1.35-1.66 |  |  | 1.37 | 1.26-1.49 |  |
| Analysis 2: baseline health^c^ & influence at work |  |  |  |  |  |  |  |
| NIE | 1.43 | 1.36-1.50 | 56 |  | 1.14 | 1.11-1.17 | 33 |
| NDE | 1.50 | 1.35-1.66 |  |  | 1.39 | 1.27-1.51 |  |
| Analysis 3: baseline health^c^ & possibilities for development |  |  |  |  |  |  |  |
| NIE | 1.43 | 1.36-1.50 | 56 |  | 1.14 | 1.12-1.16 | 33 |
| NDE | 1.50 | 1.35-1.65 |  |  | 1.39 | 1.27-1.51 |  |
| Analysis 4: baseline health^c^ & leadership quality |  |  |  |  |  |  |  |
| NIE | 1.44 | 1.37-1.51 | 57 |  | 1.13 | 1.11-1.15 | 31 |
| NDE | 1.49 | 1.34-1.65 |  |  | 1.41 | 1.29-1.52 |  |
| Analysis 5: baseline health^c^ & rewards |  |  |  |  |  |  |  |
| NIE | 1.43 | 1.36-1.50 | 56 |  | 1.13 | 1.11-1.15 | 31 |
| NDE | 1.50 | 1.34-1.65 |  |  | 1.40 | 1.28-1.52 |  |
| Analysis 6: baseline health^c^ & BMI |  |  |  |  |  |  |  |
| NIE | 1.44 | 1.37-1.51 | 58 |  | 1.19 | 1.16-1.22 | 43 |
| NDE | 1.48 | 1.33-1.64 |  |  | 1.33 | 1.22-1.45 |  |
| Analysis 7: baseline health^c^ & smoking |  |  |  |  |  |  |  |
| NIE | 1.69 | 1.57-1.82 | 76 |  | 1.17 | 1.14-1.20 | 39 |
| NDE | 1.27 | 1.11-1.42 |  |  | 1.36 | 1.24-1.48 |  |
| Analysis 8: baseline health^c^ & physical activity |  |  |  |  |  |  |  |
| NIE | 1.62 | 1.52-1.71 | 71 |  | 1.19 | 1.16-1.21 | 43 |
| NDE | 1.33 | 1.18-1.48 |  |  | 1.34 | 1.22-1.45 |  |
| ^a^obtained from bootstrapping (1 000 reps); ^b^Proportion mediated (PM) = RR_NDE_*(RR_NIE_-1)/(RR_NDE_*RR_NIE_-1)  ^c^plus partner status and working hours | | | | | | | |
