# Supplementary material for: The contribution of work and health-related lifestyle to educational inequalities in physical health among older workers in Germany. A causal mediation analysis with data from the lidA cohort study
Source: PLoS One. 2023 Aug 9;18(8):e0285319. doi: 10.1371/journal.pone.0285319 (PMC10411755; doi:10.1371/journal.pone.0285319)
Supplement: S4 Table — (DOCX) [file pone.0285319.s005.docx]

**S4 Mediational E-values for the female sub-sample to quantify the minimum strength of the association that an unmeasured confounder would need to have with both the outcome and the mediators to fully explain away the NIE.**

|  | low vs. high education | |  | moderate vs. high education | |
| --- | --- | --- | --- | --- | --- |
| Mediational E-values for NIE | RR to explain away NIE | RR to shift CI to include 1 |  | RR to explain away NIE | RR to shift CI to include 1 |
| Mediation by baseline health^a^ | 1.34 | 1.24 |  | 1.59 | 1.54 |
| Mediation by baseline health^a^ & work factors | 1.59 | 1.43 |  | 1.59 | 1.54 |
| Mediation by baseline health^a^ & work factors & health behaviours | 1.92 | 1.67 |  | 1.83 | 1.76 |
| ^a^plus partner status and working hours | | | | | |
