# Supplementary material for: The contribution of work and health-related lifestyle to educational inequalities in physical health among older workers in Germany. A causal mediation analysis with data from the lidA cohort study
Source: PLoS One. 2023 Aug 9;18(8):e0285319. doi: 10.1371/journal.pone.0285319 (PMC10411755; doi:10.1371/journal.pone.0285319)
Supplement: S1 File — (PDF) [file pone.0285319.s007.pdf]

S6 File. Stata code for mediation analysis using inverse odds weighting with imputed data; example for comparing low versus high educated men using all putative mediators

```
********************
*Use imputed data
********************
cd "C:\Users\..."
use data_imp, clear

********************
*Drop female subjects
********************
drop if (sex==1)

********************
*Overview over variables
********************
*Outcome: physical health
*Exposure: lo vs hi education
*Mediators: baseline health, partner status, working hours
// physical demands, influence at work, possibilities for development, leadership quality, rewards
// BMI, smoking, physical activity
*Confounder: age, migrant status

********************
*1) Exposure model
********************
*Insert regression of exposure on mediators and covariates,
//Mim command analyzes multiply imputed data.
mim: mlogit educ i.pcsw1di i.partnerw1 i.hoursw2 i.w2PDdi influw2_inv developw2_inv leadqualw2_inv reww2_inv
ib(first).BMIw2_kat3 ib(first).smokerw2 i.PAdiw2 i.age i.migrant, base(2)
//base(2) = hi lvl of education as reference group

*Calculate predicted log odds and use that to calculate predicted probabilities
//and inverse odds within the following program "IOWMICE"
//this is done separately for outcome levels low education (suffix 0) and medium education (suffix 1)
mim: predict logodds0, outcome(0) xb
mim: predict logodds1, outcome(1) xb

********************
*User written program
********************
cap program drop IOWMICE
program IOWMICE, rclass

*Retain estimates of predicted probability, inverse odds,
//and inverse odds weights for later use
capture drop predprob0 predprob1 inverseodds0 inverseodds1 combiW0 combiW1 wt_iow

*Exposure model was run outside program IOWMICE
*Predicted log odds were obtained outside program IOWMICE
*Use them now to calculate predicted probabilities and inverse odds;
//this is done separately for outcome levels low education (suffix 0) and medium education (suffix 1)

gen predprob0=exp(logodds0)/(1+exp(logodds0))
gen predprob1=exp(logodds1)/(1+exp(logodds1))

gen inverseodds0=((1-predprob0)/predprob0)
gen inverseodds1=((1-predprob1)/predprob1)

********************
*2) Computing IOW
********************
*Create combined weight "combiW" by multiplying longitudinal non-response weight * IOW
ge combiW0 = wt_long*inverseodds0       //for low educated
ge combiW1 = wt_long*inverseodds1       //for med educated
```

*Calculate inverse odds weights; for reference group members (i.e., people without the exposure), inverse //odds weight = the original sampling weights
//for exposed, inverse odds weight = inverse odds * original sampling weights

```
gen wt_iow = wt_long if educ==2                    //non-resp weight fore reference group (hi educ)
replace wt_iow = combiW0 if (educ==0)              //combined weight for exposed (low educated)
replace wt_iow = combiW1 if (educ==1)              //combined weight for exposed (med educated)
```

****
*3) Estimation of TE
****

*Regress outcome on exposure and confounders (sampling weight = longitudinal non-response weight)

```
mim, storebv: glm pcsw3di ib(last).educ i.age i.migrant [pweight=wt_long], fam(poisson) link(log) vce(robust)
```

//in this case determine ib(last) to set high education as reference exposure group

*Retain estimates for total effects

```
matrix bb_total= e(b)
scalar TE=bb_total[1,1]
return scalar TE=bb_total[1,1]

//the code for comparison of medium vs hi education looks the same but
//to obtain effect if exposure is 1 (medium education) from matrix
//you will need the coefficient in the first column and second row
//thus scalar [1,2], hence:
//matrix bb_total= e(b)
//scalar TE2=bb_total[1,2]
//return scalar TE2=bb_total[1,2]
//--> the same applies to retaining the direct effect a few lines below
```

****
*4) Estimation of NDE
****

*Insert the direct effect regression, which is the same as the total effect regression but applying the inverse
//odds weight (sampling weight = IOW as defined in step 2 [different for exposed and unexposed]).

```
mim, storebv: glm pcsw3di ib(last).educ i.age i.migrant [pweight=wt_iow], fam(poisson) link(log) vce(robust)
```

****
*5) Retain NDE estimates and calculate NIE (= TE - NDE)
****

*Retain estimate of direct effects. Calculate indirect effects as
//the difference between total effects and direct effects.

```
matrix bb_direct = e(b)
scalar NDE=bb_direct[1,1]
return scalar NDE=bb_direct[1,1]
return scalar NIE = TE-NDE

*matrix if education = medium (1)
//matrix bb_direct = e(b)
//scalar NDE2=bb_direct[1,2]
//return scalar NDE2=bb_direct[1,2]
//return scalar NIE2 = TE2-NDE2

end
```

****
*6) Bootstrap to obtain standard errors
****

* Request bootstrapped estimates of indirect, direct and total effects. Provide initial value of the random-
//number seed for replication. Request 1000 bootstrap replications for exponentiated coefficients.

```
bootstrap exp(r(NIE)) exp(r(NDE)) exp(r(TE)), seed(12345) reps(1000): IOWMICE
estat bootstrap, all
```

*Drop log odds obtained outside user written program “IOWMICE” before next calculation

```
drop logodds0 logodds1
```
